# Propagation characteristics of pulverized coal and gas two-phase flow during an outburst

**DOI:** 10.1371/journal.pone.0180672

**Published:** 2017-07-20

**Authors:** Aitao Zhou, Kai Wang, Lingpeng Fan, Bo Tao

**Affiliations:** 1 Beijing Key Laboratory for Precise Mining of Intergrown Energy and Resources, China University of Mining and Technology (Beijing), Beijing, China; 2 School of Resource & Safety Engineering, China University of Mining & Technology (Beijing), Beijing, China; COMSATS Institute of Information Technology, PAKISTAN

## Abstract

Coal and gas outbursts are dynamic failures that can involve the ejection of thousands tons of pulverized coal, as well as considerable volumes of gas, into a limited working space within a short period. The two-phase flow of gas and pulverized coal that occurs during an outburst can lead to fatalities and destroy underground equipment. This article examines the interaction mechanism between pulverized coal and gas flow. Based on the role of gas expansion energy in the development stage of outbursts, a numerical simulation method is proposed for investigating the propagation characteristics of the two-phase flow. This simulation method was verified by a shock tube experiment involving pulverized coal and gas flow. The experimental and simulated results both demonstrate that the instantaneous ejection of pulverized coal and gas flow can form outburst shock waves. These are attenuated along the propagation direction, and the volume fraction of pulverized coal in the two-phase flow has significant influence on attenuation of the outburst shock wave. As a whole, pulverized coal flow has a negative impact on gas flow, which makes a great loss of large amounts of initial energy, blocking the propagation of gas flow. According to comparison of numerical results for different roadway types, the attenuation effect of T-type roadways is best. In the propagation of shock wave, reflection and diffraction of shock wave interact through the complex roadway types.

## Introduction

Coal and gas outbursts are an extremely complex dynamic phenomenon[1–5], during an outburst, the coals and rocks around the coal mining face are rapidly broken and ejected, releasing large amounts of gas from the pulverized coal[6,7]. The pulverized coal and gas flow induced by an outburst have enormous energy[8], which can lead to fatalities and destroy underground equipment. In recent years, many coal and gas outburst accidents have occurred in China. For example, on October 20, 2004, a serious outburst occurred in Daping coal mine of Zheng Coal Group in Henan province. In this accident, the outburst coal and rock was estimated 1894 t, plus approximately 250 thousand m^3^ outburst gases. Because the pressure of outburst gas flow was great, some underground ventilation facilities were destroyed, a large number of gas flowed to adjacent intake roadways, such that the gas concentration within these intake roadways exceeds gas explosion limit, a gas explosion occurred. 148 people were killed and 32 people were injured.

Extensive research have been carried out on coal and gas outbursts[9–15], many models and theories have been developed, however, these achievements mainly focus on outburst mechanism and prediction as well as outburst prevention technology, little investigation has been conducted for outburst gas flows during an outburst.

Cheng et al [16] theoretical analysed the outburst shock wave formation process as well as propagation law. Otuonye et al[17]simulated outburst shock waves based on simplified outburst initiation model. A field investigation of outburst gas flow pressure was carried out in Zhongliangshan coal mine, China[18].The results showed the outburst shock wave pressures of 0.3~0.6 MPa, confirming the enormous destructive potential of outburst shock waves. Wang et al [19–21] analysed outburst shock wave propagation characteristic in different type roadways. However, none of the above researches considered the role of pulverized coal play in the propagation of outburst shock waves. During an outburst, the interaction between pulverized coal and gas flow is quite obvious.

In this study, combining with theoretical analysis and numerical simulation as well as experimental methods, the interaction mechanism between pulverized coal and gas flow was analyzed, three dimensional unsteady models for pulverized coal and gas two-phase flow was established, then the outburst pressure attenuation law was investigated.

## Methodology

### Numerical method

#### Initial conditions

Due to the complexity and variability of outbursts, it is more appropriate to analyse this phenomenon from an energy perspective. It is generally concluded that the gas expansion energy in addition to the elastic energy of coal is transferred to the coal crushing energy, the transport energy, and the remaining kinetic energy of gas after carrying the pulverized coal [22]. Zhao et al [18] calculated the elastic energy of coal and the expansion energy of gas for several outburst accidents, and showed that elastic energy only accounts for a few thousandths of the total outburst energy. Thus, in the outburst development stage, the elastic energy of coal can be ignored and the transport energy of coal derives entirely from the gas expansion energy, which can be expressed as follows:

$$W=\frac{P_{0}V_{0}}{n-1}[{\left(\frac{P_{1}}{P_{0}}\right)}^{\frac{n-1}{n}}-1]$$ (1)

where *P*_0_ and *P*_1_ are atmospheric pressure and gas pressure in the outburst hole., respectively; *n* is the adiabatic coefficient, which is usually chosen to be 1.3; *V*_0_ and *V*_1_ represent the gas volumes under pressure *P*_0_ and *P*_1_.

As shown in Eq (6), the transport energy for the two-phase flow of gas and pulverized coal is mainly dominated by the gas volume and gas pressure in the outburst hole.

**Fig 1:**
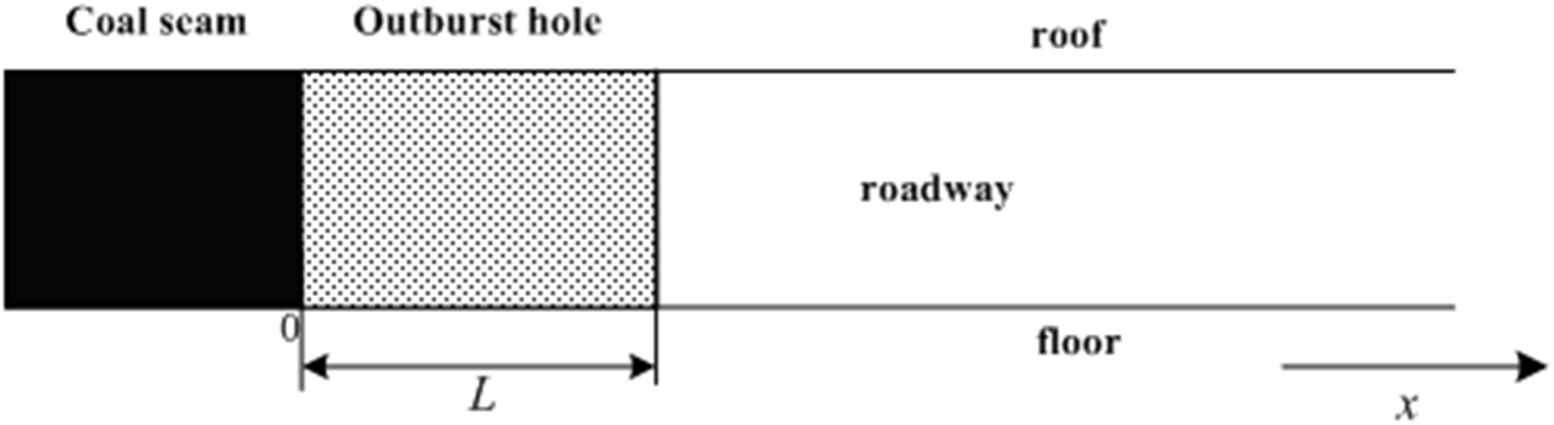
Geometric model of the roadway at the critical state during an outburst.

For numerical simulation of pulverized coal and gas two-phase flow propagation characteristics, the initial values for parameters in the simulation region need to be assumed. Fig 1 shows a geometric diagram of a coal mine roadway during the critical state of an outburst. For an outburst hole, the length is assumed to be *L*; the gas with high pressure is basically in the stationary state at the critical state of an outburst; the gas relative concentration *C*_1_ of outburst zone is 1 (assumed to be pure methane); and the temperature *T*_1_ of the gas in the outburst hole is assumed to be 300 K. Based on the energy analysis for the gas/pulverized coal two-phase flow, at the critical state, the initial condition of the gas in the outburst zone is given by Eq (2):

$$p=p_{1},u_{1}=0,T_{1}=300\text{K},\;C_{1}=1\;(t=0;\;\text{-L}<\text{x}<0)$$ (2)

Where *p* and *p*_1_ are gas pressure in the outburst hole. In general, mine roadways are arranged at a certain depth beneath the surface. There are some differences in air pressure between the roadway and surface atmospheric pressure; however, this is sufficiently small that the air pressure in the roadway is taken as the atmospheric pressure *p*_*a*_. Although airflow speed is non-zero, it does not exceed 15 m/s in the roadway and 8 m/s in the return airway. Obviously, the speed of the airflow is much slower than that of the outburst gas propagation; therefore, the airflow speed in roadways can be assumed to zero.

Gas concentration within the airflow is very low, not exceeding 1%; therefore, the volume concentration of gas in the roadway is assumed to be zero. The temperature in roadways is the same as that in the outburst hole. According to the above analysis, the initial airflow conditions in the roadways at the critical state are as follows:

$$p_{0}=p_{a},u_{0}=0,T_{1}=300\text{K},\;C_{0}=0\;(t=0;\;\text{x}>0)$$ (3)

### Experimental method

#### Experimental equipment

To validate the numerical simulation, an experimental system was constructed for simulating the propagation law of outburst shock waves [10]. Because stresses only dominate the coal elastic energy, based on the energy analysis for the pulverized coal and gas two-phase flow, the stresses can be ignored. Therefore, the experimental system can be constructed as one kind of shock tube[20].

#### Experimental procedure

(1) Coal sample preparation

A predetermined pressure is applied to the coal sample, a constant pressure process lasts about 30 minutes in order to release the gas contained in the coal sample, several times will be repeated, and then the coal sample is loaded into the outburst hole.

(2) Determining air-tightness

The outburst hole is checked for air-tightness using soapy water to detect any leaks.

(3) Coal gas adsorption. Prior to the sorption of coal gas, it is degassed to vacuum for 12 h with a vacuum pump, and then the coal is filled gas for 48 h to achieve adsorption equilibrium.

(4) After preparing the pressure transducers and data acquisition system, a much higher pressure than the adsorption equilibrium is applied to the canvas, resulting in outburst.

## Results and discussions

To analyse the influence of pulverized coal on the outburst shock waves, simulations were conducted for different volume fraction of pulverized coal and different roadway types: the volume fraction of pulverized coal is 0% indicates the pure gas outburst, while the other volume fraction of pulverized coal is 5% simulates the process of coal-gas two-phase flow. In the meantime, three different roadway types with straight, branch, bifurcation are also simulated.

### Numerical result for straight roadways

**Fig 2:**
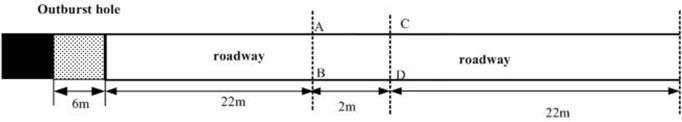
Geometry of straight roadway.

Fig 2 shows the geometry of a straight roadway with no variation in cross section. In order to reveal the attenuation law of shock wave, an observation of overpressure is done which put emphasis on cross-sections AB and CD. The gas pressure in the outburst hole is 1 MPa, two different volume fractions are chosen.

**Fig 3:**
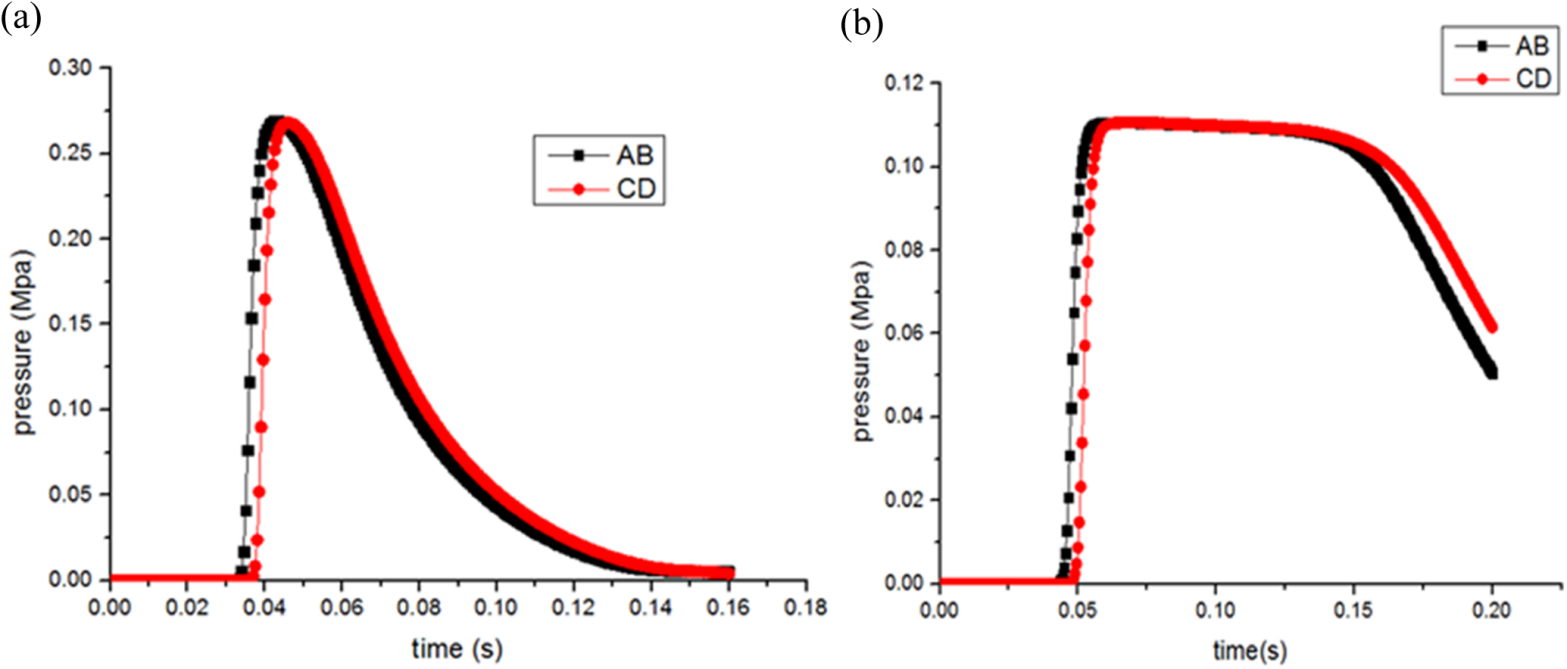
Profile of gas overpressure variation with time at different cross sections. (a) shock waves pressure(volume fraction, 0%). (b) shock waves pressure(volume fraction, 5%).

As shown in Fig 3, the peak overpressure of CD cross section appears later than that of AB section whether or not the participation of pulverized coal in the outburst process. Another explicit phenomenon need to be pointed out is that both pressure drops are minor due to the small span between two neighbor sections. In the case of pulverized coal, the peak value declines from the original 0.27 Mpa to 0.11 Mpa, arrival time of overpressure are also delayed from 0.035 s to 0.046 s, indicating that the pulverized coal flow plays a negative role which consumes large energy and blocks the gas flow. In the absence of pulverized coal, the overpressure decays more rapidly, as contrasted to the slow attenuation process once pulverized coal is consider, all that illustrates the blocking effect.

### Numerical result for T-shape roadways

**Fig 4:**
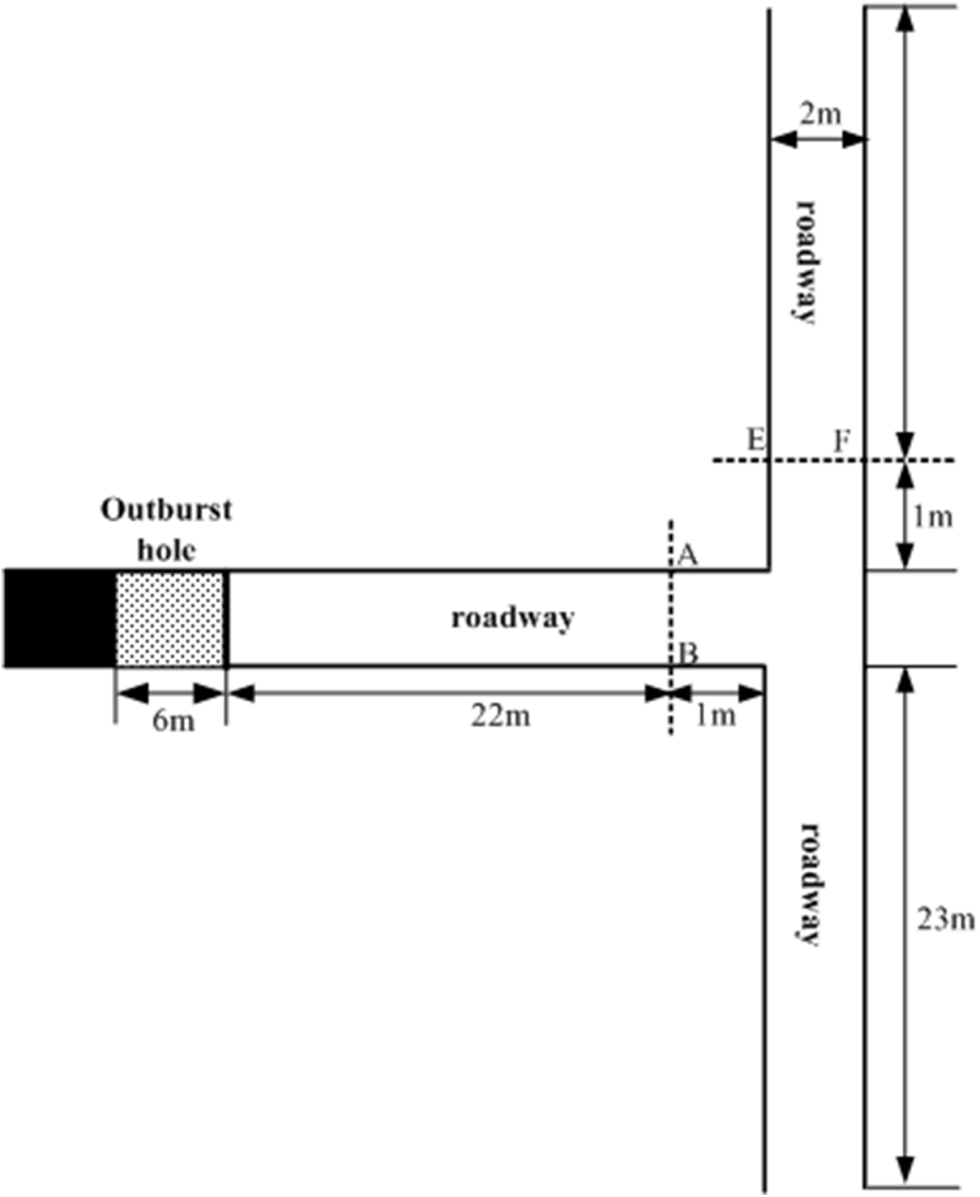
Geometry of T-shape roadway.

Fig 4 shows the geometry of a T-shape roadway, The basic size is nearly the same as that of the straight roadway, the gas pressure in the outburst hole is 1 MPa. Contrast to straight roadway, cross-sections AB and EF are marked.

**Fig 5:**
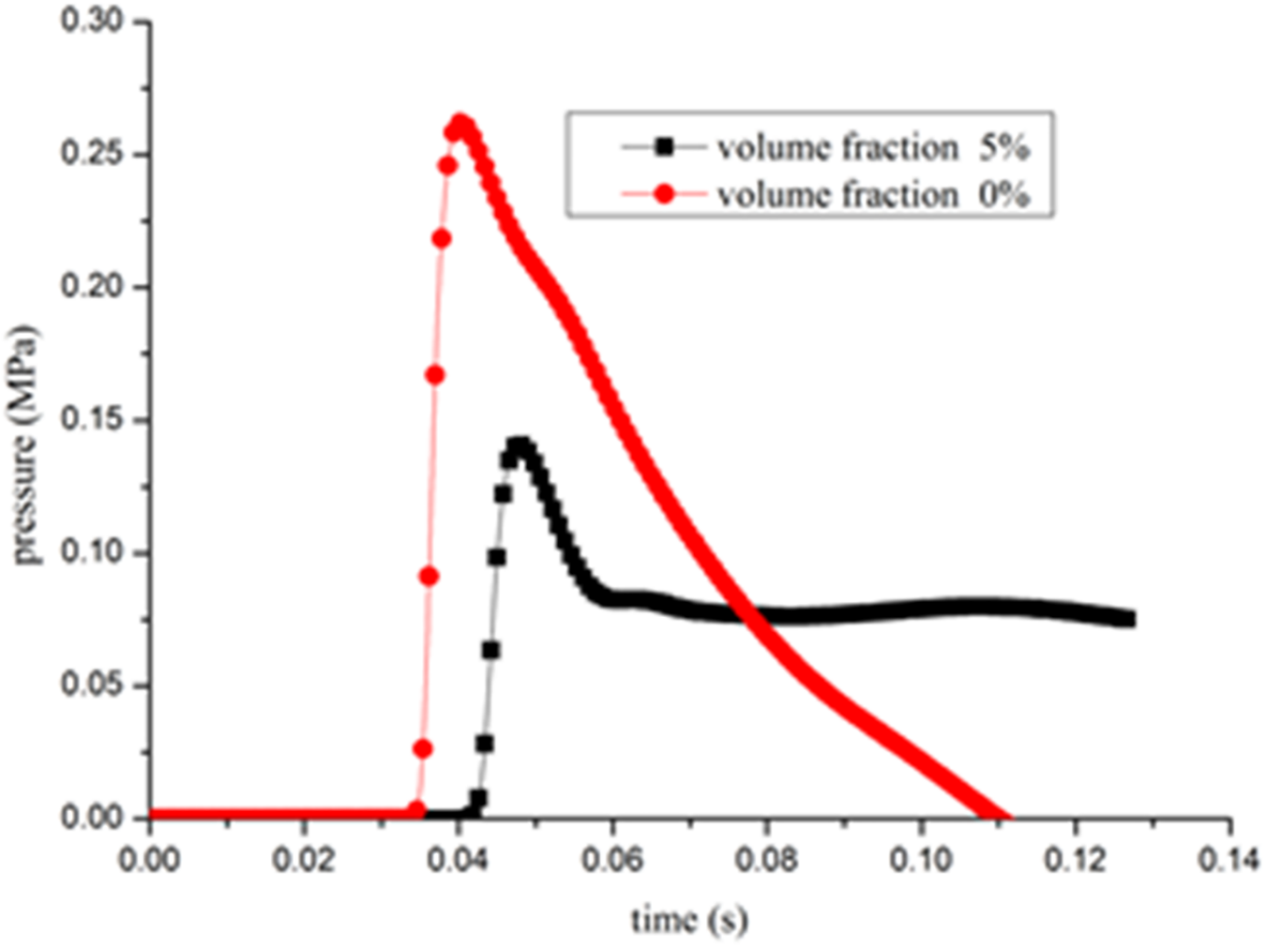
Profile of outburst shock waves pressure variation with time at AB cross section.

**Fig 6:**
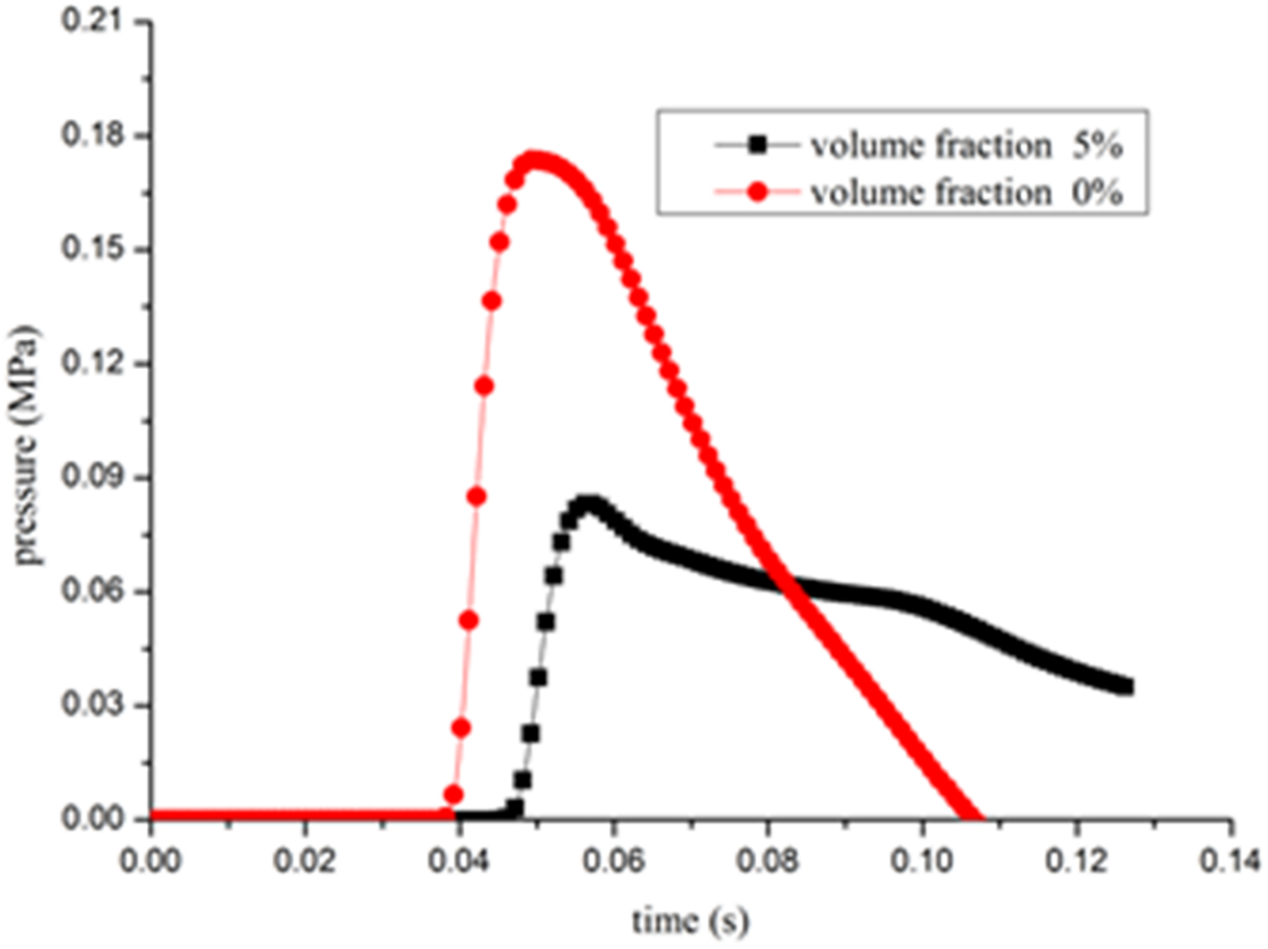
Profile of outburst shock waves pressure variation with time at EF cross section.

As shown in Fig 5 and Fig 6, outburst shock waves propagate at high speed. For a pulverized coal volume fraction of 0%, the peak overpressure of the outburst shock wave reaches cross-section AB at 0.0404 s, compared with 0.0478 s when pulverized coal volume fraction is 5%. Duo to the interaction between the pulverized coal and gas flow, part of outburst shock wave consumes energy such that the peak overpressure with pulverized coal is lower than that with no pulverized coal. Furthermore, the intensity of the outburst shock waves is attenuated along the propagation direction: For 0% pulverized coal volume fraction, the attenuation coefficient from cross-section AB to EF section is 1.86, compared with 2.22 when the pulverized coal volume fraction is 5%.

**Fig 7:**
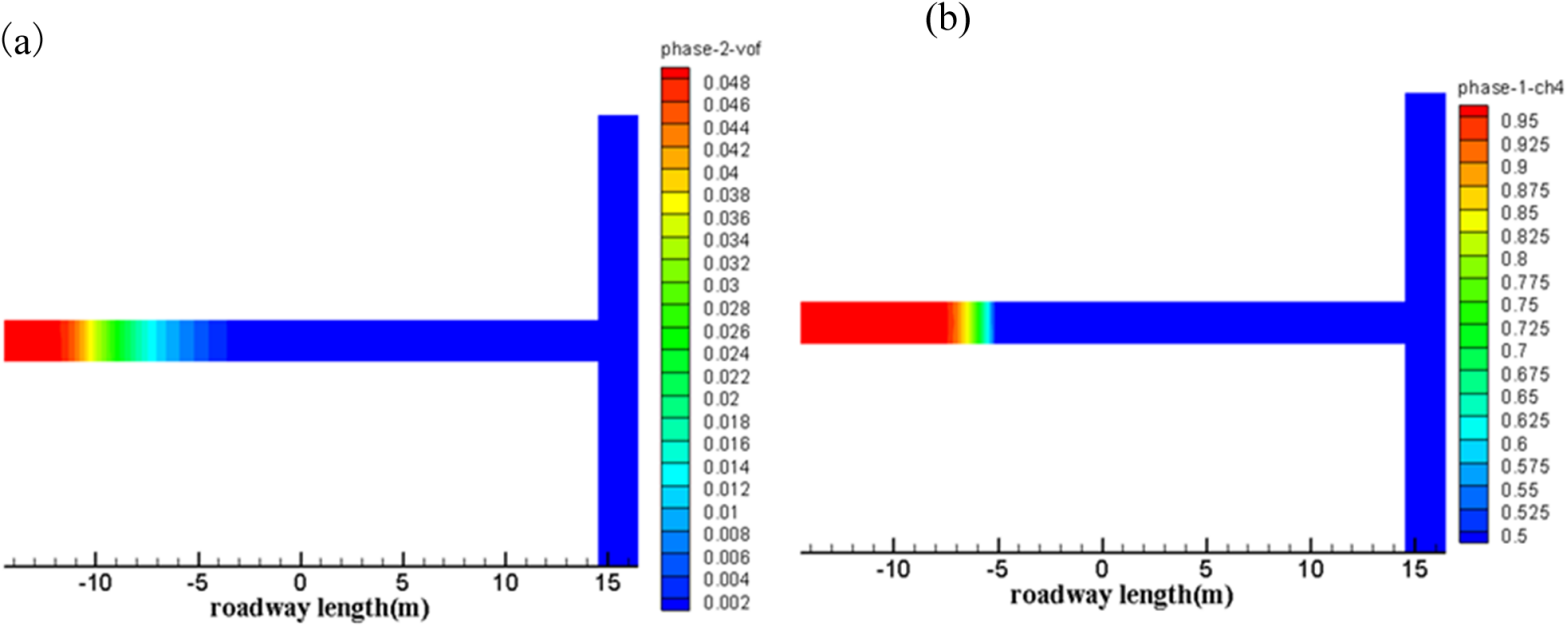
Cloud of gas and pulverized coal at 0.02 s. (a) Cloud of pulverized coal transport. (b) Cloud of gas flow migration.

Fig 7 shows propagation characteristic of the pulverized coal as well as the gas flow along the roadway direction at 0.02 s during the outbursts.

It can be seen that the transport speed of pulverized coal is much slower than that of the gas flow. At time 0.02 s, the transport distance of pulverized coal is about 5 m along the axial direction of the roadway, whereas the migration distance of gas flow is almost 8 m.

### Numerical result for bifurcation roadways

**Fig 8:**
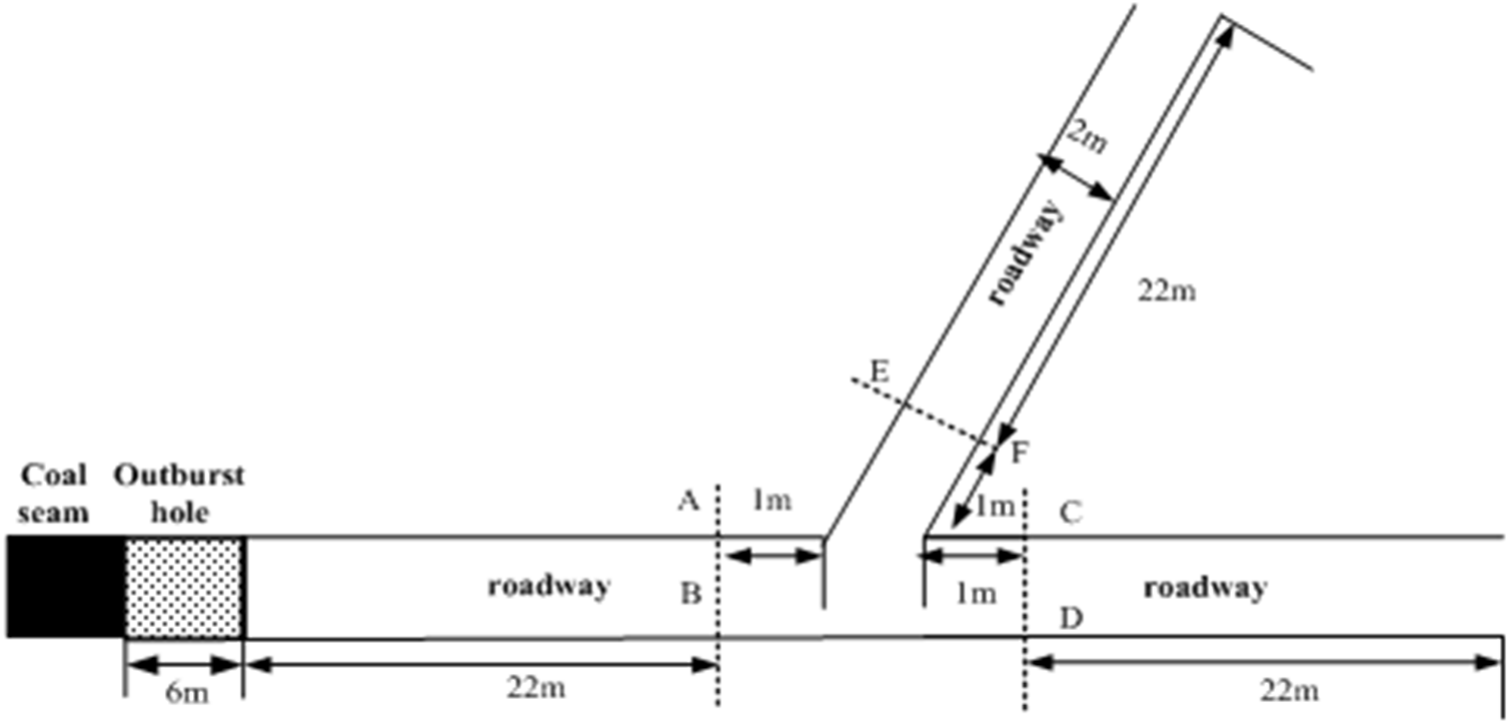
Geometry of bifurcation roadway.

Fig 8 shows the geometry of bifurcation roadway, initial parameters and scaled size are nearly the same with former condition, no more detailed information need to be given. Similarly, section AB, CD, EF are set as the monitor faces, the volume fraction of pulverized coal is 5%.

**Fig 9:**
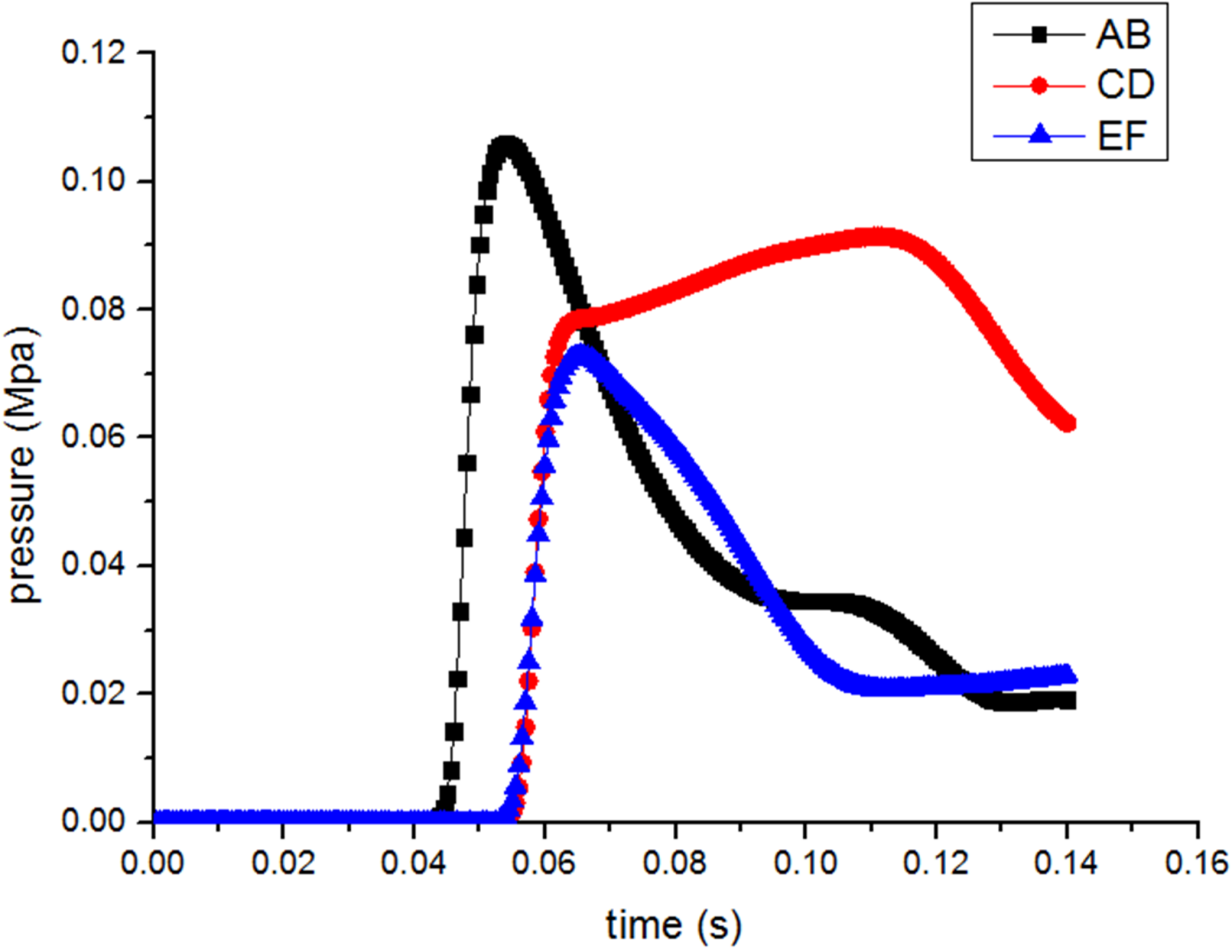
Profile of gas overpressure variation with time at different cross sections.

Fig 9 presents a comparison of the shock waves pressure variation with time at section AB, CD and EF. With the propagation of coal-gas flow, the shock wave front reaches section AB firstly, a peak overpressure appears immediately. When the shock wave passes through the bifurcation, a great pressure drop of section AB can be found in a very short time, it can be explained by the abrupt expanded area theory. In fact, in this passing process of coal-gas flow, bifurcation functions just like abrupt enlarged cross section. A small part coal-gas flow enters into the sub roadway due to the diffraction which takes place in the near-wall corner of bifurcation, while most coal-gas flow propagates by the direction of downstream main roadway. The attenuation trends of overpressure at section CD, EF exemplify above theory, peak overpressure of section CD is 0.092Mpa which is higher than section EF value of 0.073Mpa, while both smaller than upstream peak value.

### Experimental results

An experimental study on the propagation law of outburst shock waves was conducted with the experimental equipment. The initial gas pressure in the outburst hole was 0.9 MPa.

**Fig 10:**
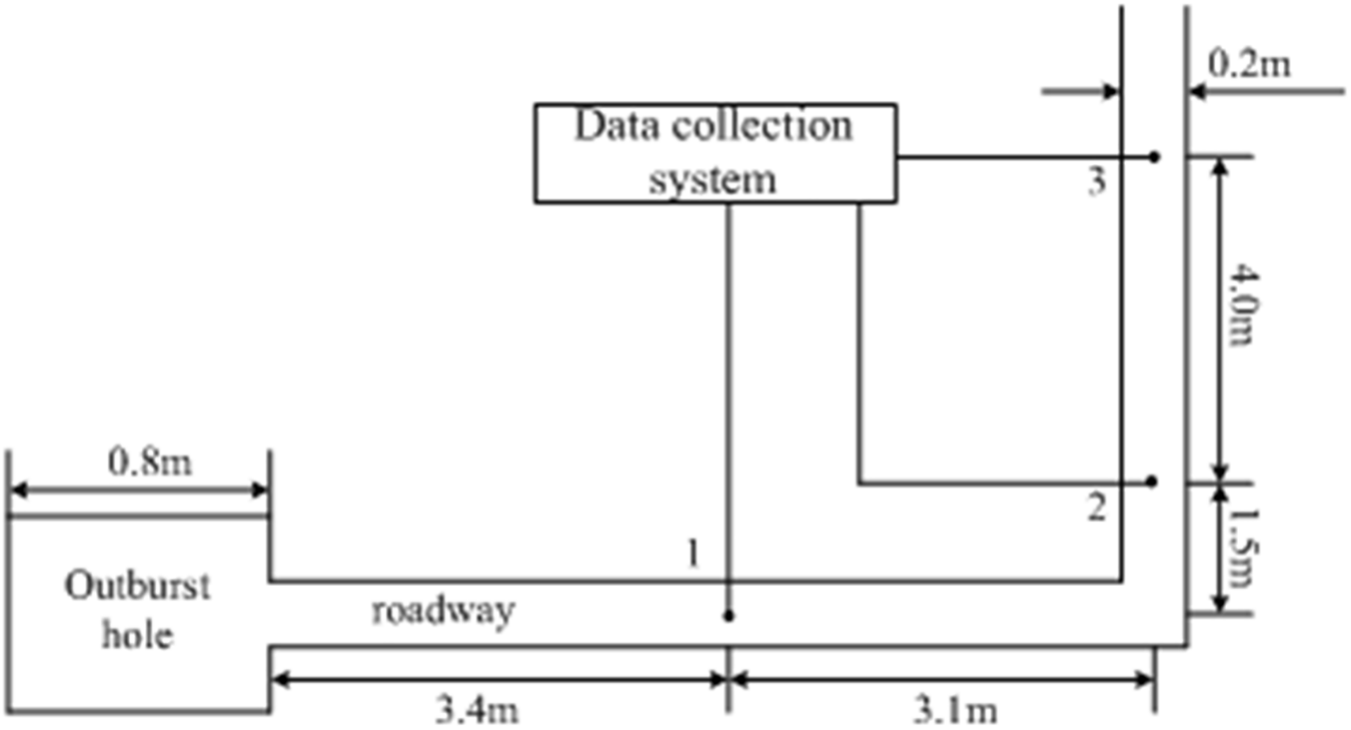
Schematic diagram of the experimental system.

As shown in Fig 10. The distance distances between the outburst hole and three pressure sensors were 3.4 m, 8.0 m, and 12.0 m, respectively. Pressure transducers were connected to the data acquisition system, and the voltage signal outputs from the sensors were collected by the acquisition system. These voltage signals were then converted into the overpressure values.

**Fig 11:**
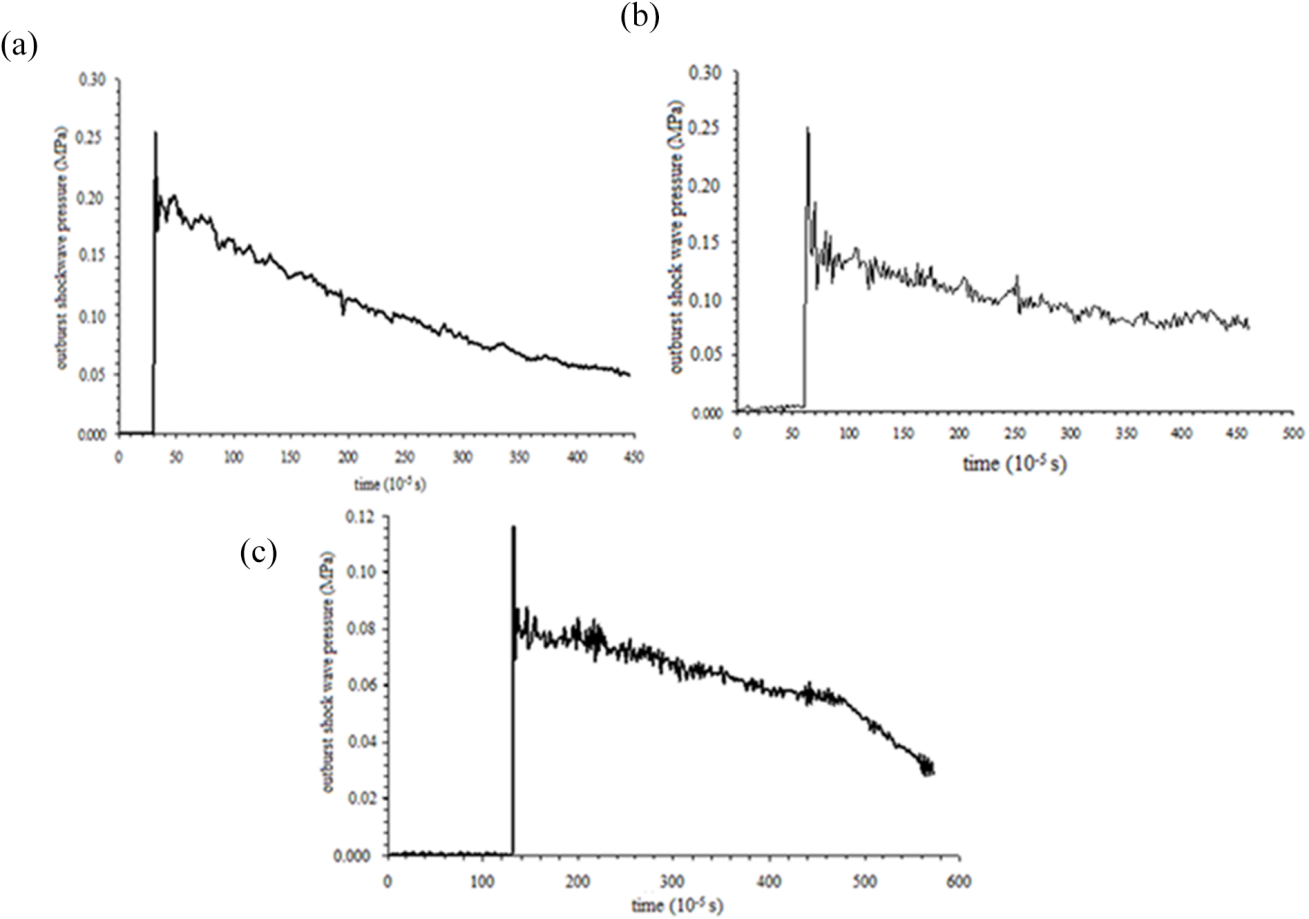
Profiles of gas overpressure variation with time at three points. (a) point No.1 (b) point No.2 (c) point No.3.

Fig 11 shows profiles of gas overpressure variation with time at three points. As shown in Fig 11, the maximum overpressures of the outburst shock waves measured at points 1, 2, and 3 are 0.255 MPa, 0.251 MPa, and 0.116 MPa, respectively. When the shock waves spread to the measuring points, the pressure changes suddenly and then decays with time, which is consistent with the results of the numerical simulation. Comparison of the maximum overpressure values between measurement points 2 and 1 shows that the pressure drop is not great. This is mainly due to shock airflow continuous diffraction at the corner, collision with the wall, and reflection.

### Numerical results for different roadway types

**Fig 12:**
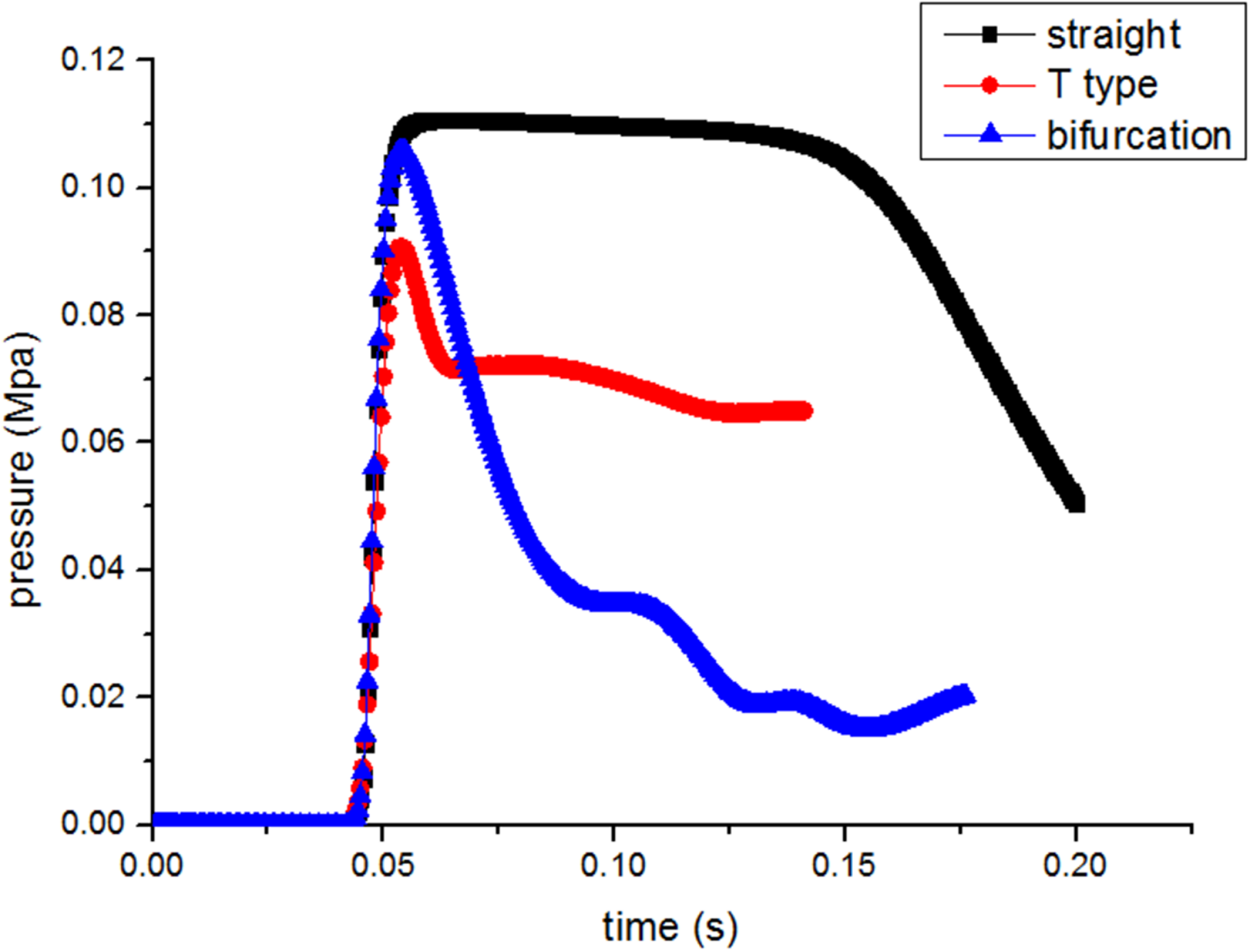
Profiles of gas overpressure at cross section AB for different roadway types.

Fig 12 shows that, compared with straight roadway, roadways of T shaped and bifurcation function well on the attenuation of over pressure, due to increased transmitted cross section of the roadway which is conducive to the rapid release of upstream coal-gas flow and pressure relief. From this perspective, in which overpressures of T-type roadways decrease most significantly, this is mainly because the transmitted shock wave front takes a strongly collision with the facing rigid wall. Conversely, the formation of the reflected wave collides with a positive transmitted shock wave, which weakens its energy, but shock wave reflection is not obvious at bifurcation roadways while the diffraction leads a prominent effect.

**Fig 13:**
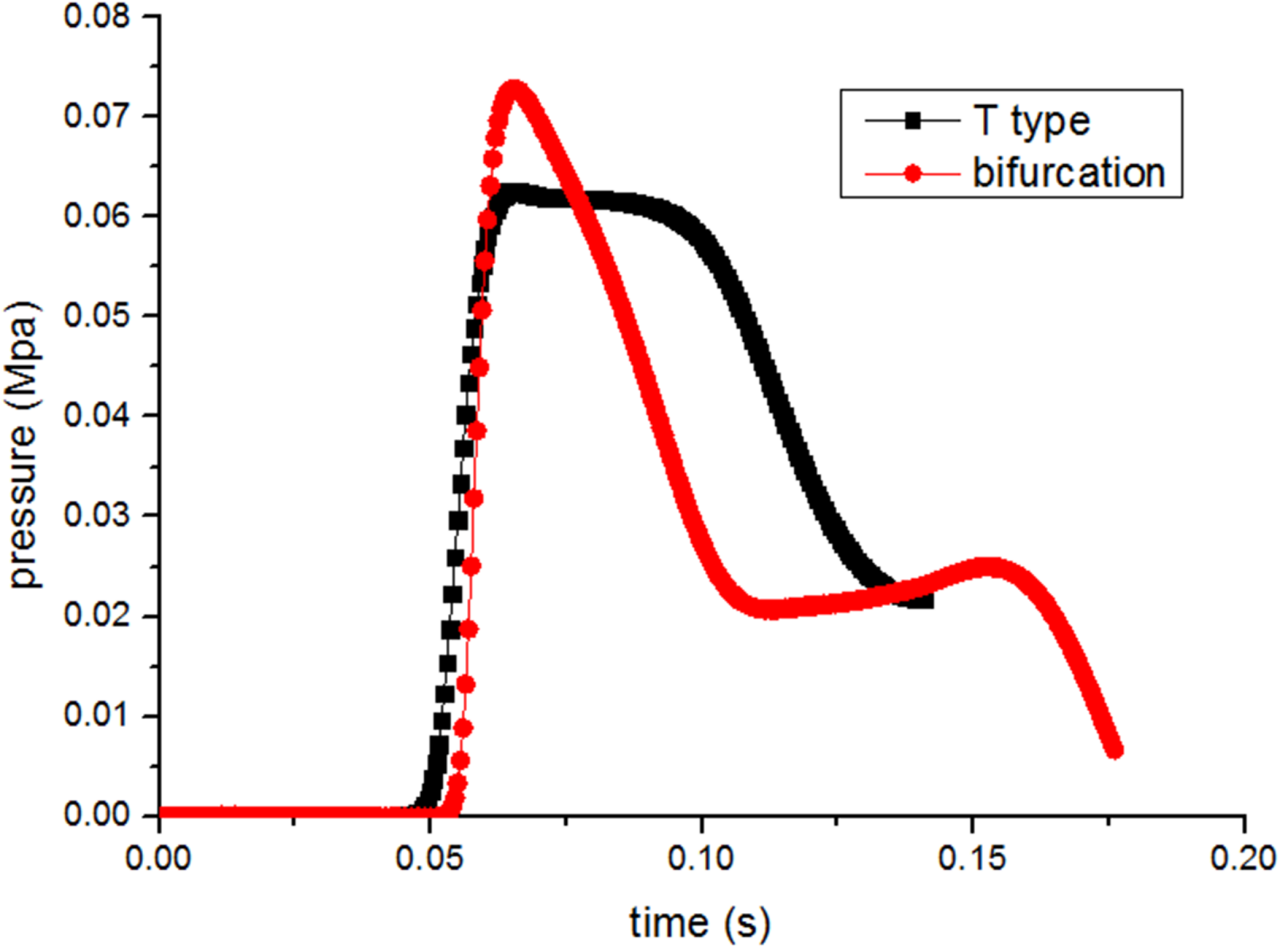
Profiles of gas overpressure at cross section EF for different roadway types.

As shown in Fig 13, shock wave mitigates in the sub roadway via different roadway types. The peak overpressure of sub roadway for T type is 0.062Mpa, while that of the bifurcation is 0.075Mpa, obviously higher than the former. It also indicates that the attenuation effect of T type is more significant which mainly contributes to the blocking effect as mentioned before. But in the subsequent decline of overpressure, bifurcation type responses rapidly, as contrast to roadways for T type. On one hand, interaction of blocking effect and collision for T type roadways makes the coal-gas flow entering into the branch slow down; on the other hand, diffraction effect of bifurcation accelerates the flow.

### Comparison of numerical results and experimental results

**Fig 14:**
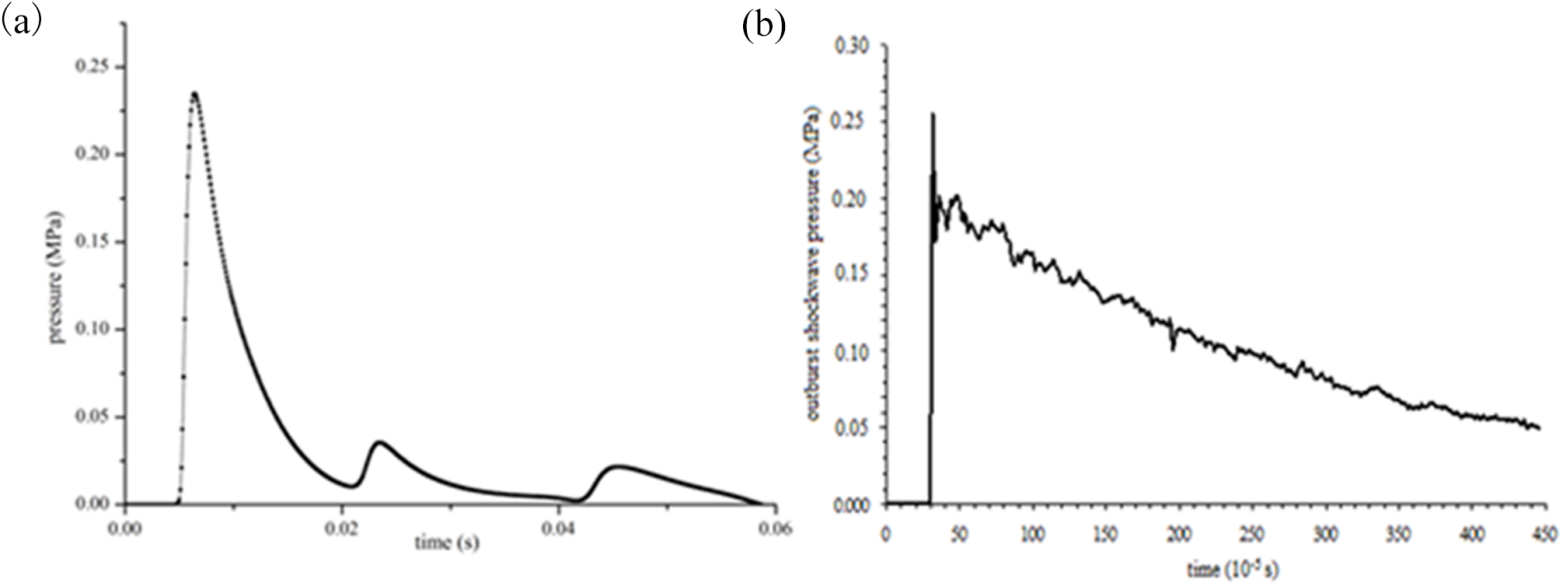
Comparison of gas overpressure at points No.1 between numerical simulation and experimental validation. (a) numerical simulation. (b) experimental result.

Based on the experiment in Fig 10, numerical simulation was conducted. Fig 14 presents the characteristics of outburst shock wave propagation. At measurement point 1, maximum overpressure is 0.231 MPa, which is approximately equal to the experimental result, and the outburst shock wave attenuation laws are similar between the numerical and experimental simulations.

## Conclusions

(1) The elastic energy of coal only accounts for a few thousandths of the total outburst energy. Thus, in the outburst development stage, the elastic energy of coal can be ignored and the transport energy of coal derives entirely from the gas expansion energy.

(2) Based on the role of gas expansion energy in the propagation characteristics of pulverized coal and gas two-phase flow during an outburst, a numerical simulation method and an experiment system were constructed for revealing the attenuation law of this two-phase flow.

(3) Pulverized coal and gas at high pressure are instantly ejected from the outburst hole; rapidly inflate; and compress the air in roadway, thereby producing outburst shock waves. Outburst shock waves rapidly propagate in an axial direction along the roadway.

(4) The outburst shock wave induced by the pulverized coal and gas flow attenuates along the roadway in an axial direction, and the volume fraction of pulverized coal plays important role in the attenuation of the outburst shock wave.

(5) Compared with straight roadway, roadways of T shaped and bifurcation function well on the attenuation of over pressure, in which overpressure of T-type roadways decreases most significantly, Abrupt expanded area theory can be applied into the explanation of this phenomenon. For T type roadways, interaction of blocking effect and collision play the leading part in the propagation of shock wave, while the diffraction effect is dominant in the bifurcation roadways.

## Abbreviations

${\vec{v}}_{m}$: Mass-averaged velocity (m/s)
*t*: Time (s)
*h*: Enthalpy (KJ/kg)
*T*: Temperature (K)
*E*_*t*_: Total energy of airflow (J)
*μ*: Dynamic viscosity kg/(m·s)
*γ*: Adiabatic coefficient
*ρ*: Airflow density (kg/m^3^)
*a*_*k*_: Volume fraction of phase *k*
*n*: Number of phases
*C*_*P*_: Specific heat at constant pressure J/(kg·K)
*C*_0_: Local sonic speed (m/s)
*m*: Mixture (-)
*k*: Phase *k*. (-)
